# A 3D high resolution MRI method for the visualization of cardiac fibro-fatty infiltrations

**DOI:** 10.1038/s41598-021-85774-6

**Published:** 2021-04-29

**Authors:** K. Haliot, V. Dubes, M. Constantin, M. Pernot, L. Labrousse, O. Busuttil, R. D. Walton, O. Bernus, J. Rogier, K. Nubret, P. Dos Santos, D. Benoist, M. Haïssaguerre, J. Magat, B. Quesson

**Affiliations:** 1 IHU L’Institut de RYthmologie et de Modélisation Cardiaque (LIRYC), Electrophysiology and Heart Modeling Institute, Fondation Bordeaux Université, 33600 Pessac-Bordeaux, France; 2 Centre de recherche Cardio-Thoracique de Bordeaux, U1045, Université de Bordeaux, 33000 Bordeaux, France; 3 INSERM, Centre de Recherche Cardio-Thoracique de Bordeaux, U1045, Université de Bordeaux, 33000 Bordeaux, France; 4 Bordeaux University Hospital (CHU), 33600 Pessac, France

**Keywords:** Cardiology, Medical research

## Abstract

Modifications of the myocardial architecture can cause abnormal electrical activity of the heart. Fibro-fatty infiltrations have been implicated in various cardiac pathologies associated with arrhythmias and sudden cardiac death, such as arrhythmogenic right ventricular cardiomyopathy (ARVC). Here, we report the development of an MRI protocol to observe these modifications at 9.4 T. Two fixed ex vivo human hearts, one healthy and one ARVC, were imaged with an Iterative decomposition with echo asymmetry and least-square estimations (IDEAL) and a magnetization transfer (MT) 3D sequences. The resulting fat fraction and MT ratio (MTR) were analyzed and compared to histological analysis of the three regions (“ARVC triangle”) primarily involved in ARVC structural remodeling. In the ARVC heart, high fat content was observed in the “ARVC triangle” and the superimposition of the MTR and fat fraction allowed the identification of fibrotic regions in areas without the presence of fat. The healthy heart exhibited twice less fat than the ARVC heart (31.9%, 28.7% and 1.3% of fat in the same regions, respectively). Localization of fat and fibrosis were confirmed by means of histology. This non-destructive approach allows the investigation of structural remodeling in human pathologies where fibrosis and/or fatty tissue infiltrations are expected to occur.

## Introduction

Various cardiac arrhythmias, such as atrial fibrillation, ventricular tachycardia and ventricular fibrillation can be caused and maintained by alterations in the tissue architecture and intercellular coupling, through for example fibro-fatty infiltrations^1^. In the context of atrial fibrillation, structural remodelling initially develops as a consequence of the electrical, mechanical and hemodynamical disturbances, but becomes, as the pathology progresses, a key determinant in arrhythmia complexity and therapeutic success. Recent studies using high resolution MR imaging on samples of explanted human atria revealed significant fatty infiltrations and fibrotic tissue^2^. Excessive tissue remodelling, such as the accumulation of intramuscular adipose tissue in arrhythmogenic right ventricular cardiomyopathy (ARVC), can cause ventricular tachy-arrhythmias and sudden cardiac death^1,3^. More recently, subtle localized tissue alterations have been implicated in arrhythmogenesis and sudden cardiac death in patients suffering from primary electrical disorders or idiopathic ventricular fibrillation^4,5^. Although histology performed on a limited number of biopsies have shown fibro-fatty tissue infiltrations, these tissue alterations remain undetectable with current imaging approaches and risk prediction remains poor.

In the case of ARVC, arrhythmia originate pre-dominantly from the right ventricle (RV)^6^. In this pathology, structural modifications mainly appear in the RV inflow tract, the outflow tract (RVOT) and apex of the RV; these are regarded as the “ARVC triangle” or “triangle of dysplasia”^7,8^. RV myocardial scarring initially produces regional wall motion abnormalities, but later may involve the free wall and become global; it may then produce RV dilatation and myocardial thinning. Tissue replacement can also occur in the left ventricle (LV) in the lateral and posterior walls; this is characterized by myocyte loss and fibrosis with relative sparing of the septum^9,10^. Fat infiltrates the free wall of the RV and the apex of the LV, such that both ventricles exhibit similar amounts of fat^11,12^. Clinical diagnosis of this pathology relies mainly on imaging assessment of cardiac wall kinetics, combined with histological analysis of local endocardial biopsy of the RV and the study of the genetic background of the pathology^13,14^; these together provide a limited description of the 3D extent of fibro-fatty infiltrations. However, for precise visualization of fat and fibrosis in the RV, current spatial resolution of clinical MR images remains limited^15^.

Several MRI techniques have been developed to separate fat and water in cardiac tissue in vivo^16,17^, including the use of chemical shift saturation^18^, longitudinal relaxation time weighted images^19^, and multi-echo methods such as the Dixon technique^20^. Reeder et al.^21,22^ developed iterative decomposition with echo asymmetry and least-square estimations (IDEAL) imaging to separate water and fat with chemical shifts; this approach exhibits better robustness against field inhomogeneity, compared to the conventional Dixon technique^20^.

The magnetization transfer (MT) technique was proposed to visualize fibrosis in vivo and ex vivo in liver^23^, kidney^24,25^, and intestinal tissue^26^; this contrast mechanism is sensitive to collagen content, as confirmed by histological analysis.

The objective of this study was to develop and evaluate a set of high-resolution 3D MRI acquisition sequences to localize and characterize fatty infiltrations and fibrosis in ex vivo formalin-fixed human hearts. Two hearts were included in the study to assess the specificity of the protocol. One from a patient with ARVC, where fibro-fatty infiltrations are expected, and a heart from a patient without known cardiac pathology. We present 3D images of the hearts obtained at 9.4 T from the MT^26^ sequences, the IDEAL sequence^22^, and the hierarchical IDEAL^27^ processing at an isotropic resolution of 200 µm. MRI results were analyzed and compared with those of histological analysis in regions corresponding to the ARVC triangle.

## Methods

### Sample preparation

Two human hearts were included in this study. An ex vivo human donor heart, dimensions 13 × 10 × 9.6 cm^3^, was obtained from a 36-year-old man with known ARVC undergoing cardiac transplantation at Bordeaux University Hospital. This patient was included in a heart donor program before heart transplant. Diagnosis of ARVC resulted from echography examination (dysplasia and RV dilation, RV systolic dysfunction and flattening of intraventricular septum were observed, 48% of LV ejection fraction were evaluated). A second ex vivo human donor heart, with dimensions 13.9 × 8.1 × 7.9 cm^3^, was obtained from an 83 years-old patient through a human organ donor program at the Bordeaux University Hospital. No significant cardiac pathology was revealed during the clinical evaluation performed before explanting the heart: cardiac function was preserved without diastolic dysfunction, normal right and left pressures, a small mitral regurgitation without left atrium dilation was observed and no other valvulopathy was identified. This heart was thus labelled “healthy” in the rest of the manuscript. Both hearts were fixed with 1 L formalin 10% containing 2 ml gadolinium Dotarem, by means of retrograde perfusion from the aorta at 25 rpm for 48 h. They were continuously mechanically agitated in the fixative solution for 2 days at 4 °C, then stored in the same solution at 4 °C.

For MRI acquisition, both the hearts were removed from the solution a few minutes before scanning to allow formaldehyde to evaporate under a ventilation hood; the hearts were then immersed in Fomblin (Aldrich) inside a plastic container. Fomblin is a perfluoropolyether with no detectable ^1^H MR signal, which has magnetic susceptibility similar to that of the tissue; it is used to attenuate susceptibility artefact at the borders of cardiac chambers^28^.

### MRI acquisition

All experiments were performed using a 9.4 T in a horizontal open-bore access of 30 cm (Bruker BioSpin MRI, Ettlingen, Germany). A 7-channel transmission/reception array coil (165 mm inner diameter) and a B-GA20S gradient insert of 200 mm inner diameter (1040 T m^−1^ s^−1^ maximum linear slew rate and 300 mT m^−1^ maximum gradient strength) were used to image the cardiac samples.

#### Water-fat imaging

An IDEAL^22^ gradient echo sequence was implemented in ParaVision 6.0 (Bruker BioSpin MRI, Ettlingen Germany). Acquisition parameters were: repetition time (TR) = 30 ms; echo time (TE) = 3.08/3.31/3.54 ms; number of averages (NA) = 4; flip angle = 17°; field of view (FoV) = 125 × 110 × 110 mm^3^; matrix = 625 × 550 × 550 resulting in an isotropic resolution of 200 µm; bandwidth = 250 kHz; and GRAPPA = 2 in the Y phase encoding direction, for a total acquisition time of 9 h.

#### Fibrosis imaging

The MT 3D data set (MT_on_) was implemented using a modified fast low angle shot sequence. The MT preparation module consisted of 60 hard pulses of 5 ms each, with 10 µs interpulse delay (303 ms total duration), a B_1_ amplitude of 10 µT, and a frequency offset of 3 kHz. Acquisition parameters were TR/TE = 2000 ms/9 ms, NA = 1, flip angle = 90°, FoV = 125 × 110 × 110 mm^3^, matrix = 312 × 275 × 275 resulting in an isotropic resolution of 400 µm, bandwidth = 250 kHz, and GRAPPA = 2 in the Y phase encoding direction. A reference 3D volume was acquired with identical parameters, without the MT preparation module (MT_off_). Each acquisition required 22 h 45 min.

#### Acquisition protocol

After each sample had been positioned in the magnet, scout view images were obtained to center the 3D volume, based on the ventricles. First, volumetric B_0_ shimming was performed using the tool provided by the manufacturer to achieve a full width at half maximum < 100 Hz. Before acquisition of 3D water-fat and MT images, a rapid acquisition with relaxation enhancement (RARE) sequence was used to determine T_1_ relaxation time. For this, the saturation recovery technique was used with varying TR to collect a series of T_1_-weighted images. Acquisition parameters were 6 TR arrays = 200, 400, 800, 1500, 5000, and 10,000 ms; TE = 30.6 ms; FoV = 110 × 110 mm^2^; matrix = 275 × 275; slice thickness = 2 mm; RARE factor = 8; and bandwidth = 50 kHz for a total acquisition time of 10 min 15 s. A multiple gradient echo (MGE) sequence with 8 echoes was used to measure T_2_^*^ in the same slice. Acquisition parameters were TR = 100 ms, NA = 1, FoV = 110 × 110 mm^2^, matrix = 275 × 275, slice thickness = 2 mm, and bandwidth = 100 kHz; the first TE began at 4 ms with an echo spacing of 5 ms, for a total acquisition time of 27 s.

3D water-fat images were acquired, followed by MT_off_ and MT_on_ images using the parameters described above.

### Post processing

All the MR data were fully reconstructed using ParaVision 6.0 (Bruker BioSpin MRI, Ettlingen Germany). T_1_ and T_2_^*^ maps were calculated based on the RARE and MGE datasets with the built-in Paravision 6.0 image data processing by doing pixel-wise exponential fittings:

1 $$S\left(TR\right)=k{S}_{0}(1-{e}^ {{-TR} \mathord{\left/ {\vphantom {{-TR} {T_{1} }}} \right. \kern-\nulldelimiterspace} {T_{1} }})$$

2 $$S\left(TE\right)=k{S}_{0}{e}^{{-TE}\mathord{\left/{\vphantom {{-TR} {T_{2}^{*} }}} \right. \kern-\nulldelimiterspace}{T_{2}^{*} }}$$

The means of T_1_ and T_2_^*^ for fat and cardiac tissues were estimated from a selected region of interest (ROI) in the tissue and in the pericardial fat.

#### Processing of IDEAL images

For the IDEAL sequence, a complex 3D image was reconstructed for each coil element and each echo. These images were post-processed in Matlab (MATLAB 9.5, Mathworks, Inc., Natick, MA, USA) as follows:

For each echo, the magnitude image was produced by a root-mean-squared over the different coils; the phase image was reconstructed by addition of the phase of each coil element for each pixel. The relative phase shift between each echo was then computed by subtraction of the phase of the shortest echo. Water and fat 3D images were then reconstructed from this dataset using the hierarchical IDEAL algorithm^27^. Proton density images of water (M_w_) and fat (M_f_) were computed from water and fat using the following formulas, where α is the flip angle, T_1w_ and T_2_^*^_w_ were relaxation times of cardiac tissue, while T_1f._ and T_2_^*^_f_ were relaxation times of adipose tissue.

3 $$\left\{ {\begin{array}{*{20}c} {M_{w} = W\frac{{1 - \cos \alpha e^{{{\raise0.7ex\hbox{${ - TR}$} \!\mathord{\left/ {\vphantom {{ - TR} {T_{1w} }}}\right.\kern-\nulldelimiterspace} \!\lower0.7ex\hbox{${T_{1w} }$}}}} }}{{\sin \alpha \left( {1 - e^{{{\raise0.7ex\hbox{${ - TR}$} \!\mathord{\left/ {\vphantom {{ - TR} {T_{1w} }}}\right.\kern-\nulldelimiterspace} \!\lower0.7ex\hbox{${T_{1w} }$}}}} } \right)}}e^{{{\raise0.7ex\hbox{${TE}$} \!\mathord{\left/ {\vphantom {{TE} {T_{2w}^{*} }}}\right.\kern-\nulldelimiterspace} \!\lower0.7ex\hbox{${T_{2w}^{*} }$}}}} } \\ {} \\ {M_{f} = F\frac{{1 - \cos \alpha e^{{{\raise0.7ex\hbox{${ - TR}$} \!\mathord{\left/ {\vphantom {{ - TR} {T_{1f} }}}\right.\kern-\nulldelimiterspace} \!\lower0.7ex\hbox{${T_{1f} }$}}}} }}{{\sin \alpha \left( {1 - e^{{{\raise0.7ex\hbox{${ - TR}$} \!\mathord{\left/ {\vphantom {{ - TR} {T_{1f} }}}\right.\kern-\nulldelimiterspace} \!\lower0.7ex\hbox{${T_{1f} }$}}}} } \right)}}e^{{{\raise0.7ex\hbox{${TE}$} \!\mathord{\left/ {\vphantom {{TE} {T_{2f}^{*} }}}\right.\kern-\nulldelimiterspace} \!\lower0.7ex\hbox{${T_{2f}^{*} }$}}}} } \\ \end{array} } \right.$$

From these images, the Proton Density Fat Fraction 3D map (PDFF)^29^ was calculated using the following formula:

4 $$PDFF=\frac{{M}_{f}}{{M}_{w}+{M}_{f}}\times 100$$

A 3D mask was generated to remove pixels with low intensity to analyze the fat content in each region of the heart. The square root of the sum of squares of the magnitude of the 3 IDEAL echoes was computed; a 3D mask was initially generated by using a threshold equal to three times the standard deviation of the noise^30^ in a manual ROI drawn in a central slice outside of the heart. This mask was then corrected to include pixels at the external border of pericardial fat, using a level tracing segmentation tool available in 3D Slicer^31^ (www.slicer.org). A final manual correction was performed on each slice to remove potential residual pixels.

This mask was applied to the 3D PDFF and a cumulative histogram with 1000 bins was computed. This histogram was fitted with a Gaussian mixing model, initialized with 4 peaks corresponding to water (peak with highest intensity on the histogram) and fat (peak with second highest intensity on the histogram) signals and residuals on the histogram. The upper bin value corresponding to 99.99% of the first Gaussian function was selected as the threshold to identify voxels containing water alone. A second threshold was defined using the classic Dixon water-fat separation technique, in which voxels > 50% were tagged as fat and voxels < 50% were tagged as water^20^. 3D renderings were processed using VolView (Kitware, Clifton Park, NY, USA).

#### Processing of magnetization transfer images

K-spaces of fast low angle shot acquisitions with (MT_on_) and without transfer module (MT_off_) were zero-filled by a factor of 2 before fast Fourier transformation to match the IDEAL image resolution. Then, the magnetization transfer ratio (MTR) map was reconstructed as follows$:$

5 $$MTR=\frac{{MT}_{on}}{{MT}_{off}} \times 100$$

### Histology

The pathological heart was dissected to assess the presence of fat and fibrosis. Three samples were extracted from regions of the ARVC triangle, with approximate dimensions of 1.4 × 1 × 0.6 cm^3^ for the RVOT, 2 × 1.5 × 1 cm^3^ for the RV free wall, and 1.9 × 1.8 × 1.5 cm^3^ for the RV apex with junction to the septum (RV apex septum). Note that RV apex septum sample contains both the RV apex and tissue from the LV. Following the same protocol, the healthy heart was also dissected with approximate dimensions of 1.4 × 1.1 × 1.2 cm^3^, 2 × 1.7 × 1.1 cm^3^ and 1.7 × 2.4 × 1.1 cm^3^ respectively.

Samples were first rinsed in phosphate-buffered saline, then agitated at 4 °C for 24 h. After samples had been dehydrated, each sample was embedded in paraffin and sectioned at 6 µm in the transmural direction. Tissue sections were stained with Masson’s trichrome^32^ for structural identification: collagen fibers in tissues were green; nuclei were black; and myocytes were red/pink. Slices were examined at 10x magnification on a scanner Axio Scan Z1 (Carl Zeiss SAS, Marly le Roi, France). Images were visualized using Zen lite software (ZEN Blue 2.6, Carl Zeiss Microscopy, Thornwood, NY, USA).

The fat quantification was performed by thresholding the RGB color histogram of the histological slices using ImageJ software (NIH)^33^. The analysis was performed by the same person to avoid inter-observer variability. The resulting masks were applied to count the number of pixels attributed to fat and to the cardiac tissue. The fat ratio was then computed for each slice of a given region and averaged (mean ± SD). The number of slices in ARVC and healthy hearts were (9, 10, 17) and (8, 5, 8) for the RV free wall, RV apex and RVOT, respectively.

### Selection of regions of interest on MR images

To study the ARVC triangle in both hearts, identical 3D boxes were drawn on the PDFF map, MT images, and MTR map. These boxes surrounding the RVOT, the RV free wall and the RV apex septum were 1.6 × 2.2 × 1 cm^3^, 2.6 × 2 × 2.6 cm^3^, 2.2 × 1.4 × 2.3 cm^3^, respectively. Their dimensions were chosen to include the volume of the samples based on the histology of the ARVC heart and were applied identically on both hearts. Anatomical landmarks for positioning the boxes were defined as follow: for the RVOT, the upper bound of box was chosen under the pulmonary valve; for RV free wall the upper bound of box was placed under the lower bound of the RVOT box; for the RV apex septum, the lower bound of box was chosen at the junction between the RV wall and the apex region of the septum. Within these boxes, we computed the total number of pixels included into the 3D mask (volume of tissue) defined above and the relative content of water and fat.

### Ethics approval and consent to participate

All experiments were conducted in accordance with the declaration of Helsinki. The two hearts included in this study were obtained through two research programs: The heart from the patient suffering from ARVC was obtained in the context of heart transplantation by the University Hospital of Bordeaux. The heart was collected as a human biological sample resulting from standard care activities and dedicated to scientific research (program "ARMONICA" approved by University Hospital of Bordeaux), after obtaining written informed consent from the patient. This program did not require additional approval by external review board. The heart from the 83 y.o. patient was obtained through the Human donor program "CADENCE" (providing access to heart samples from patients under cerebral death for scientific research purposes) approved by the French "Agence de la Biomédecine". After obtaining written informed consent from the patient's family, the heart was collected as a human biological sample for scientific research.

None of the experiments involved hearts procured from prisoners.

## Results

### Water-fat separation and magnetization transfer of the whole heart

**Figure 1:**
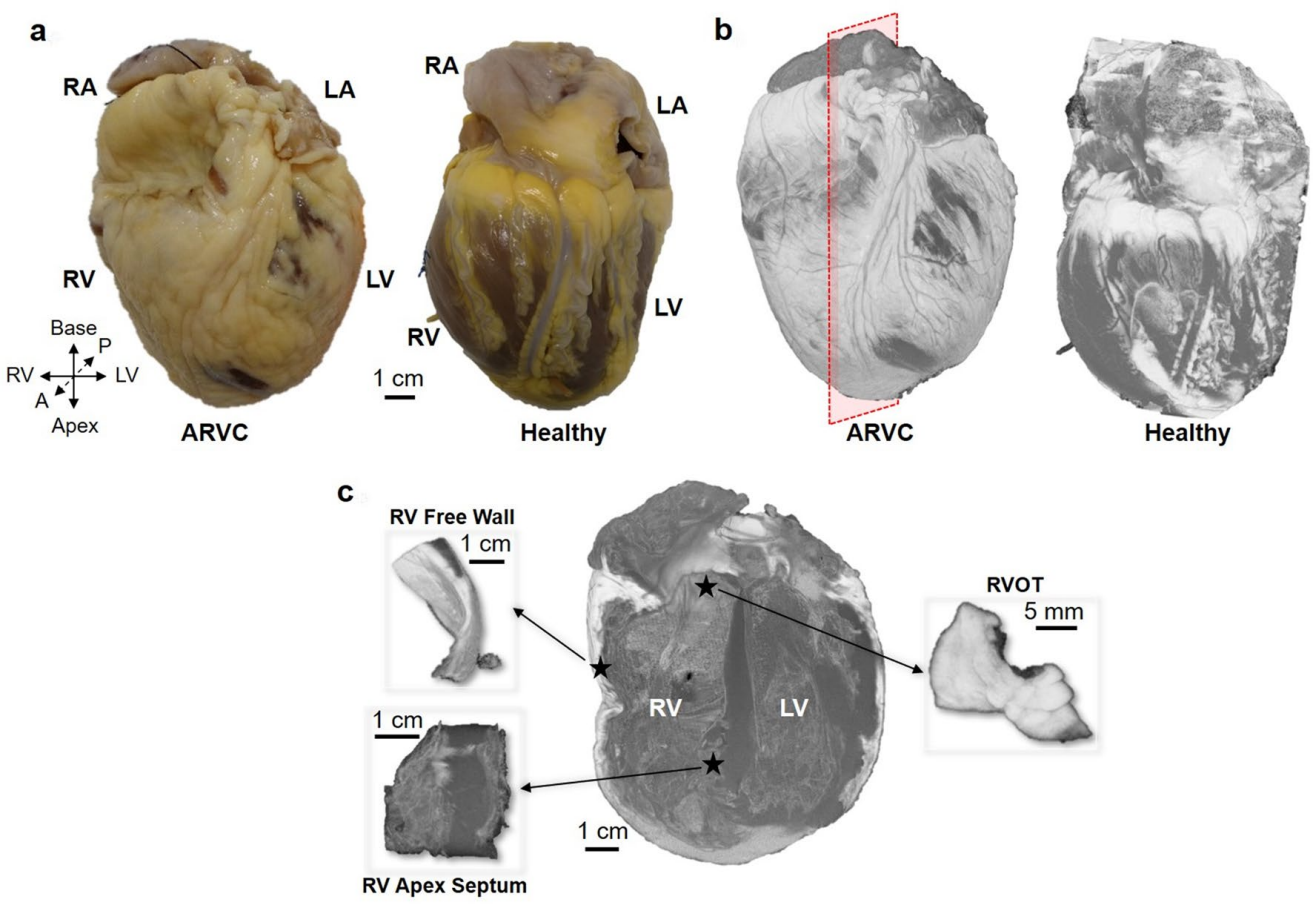
Presentation of the ARVC and healthy hearts. Photograph (**a**) of the ARVC heart (dimensions 13 × 10 × 9.6 cm^3^) and of the healthy heart (dimensions 13.9 × 8.1 × 7.9 cm^3^). (**b**) 3D reconstructions of both hearts from the fat fraction maps computed from high resolution MR images. Red plane in image on the left corresponds to the long axis plane. (**c**) ARVC heart is cropped in long axis view (red plane in **b**) to show the three sub-regions of “ARVC triangle”: the right ventricle (RV) free wall, the RV apex septum and the right ventricle outflow tract (RVOT).

Figure 1a shows two photographs of the fixed human hearts, in which tissue appearing in yellow corresponds to adipose tissue for both the ARVC (Fig. 1a left) and the healthy (Fig. 1a right) hearts. Figures 1b is a 3D rendering of the PDFF that resulted from processing of the IDEAL dataset of the two hearts; bright voxels correspond to fat. Both the photograph and 3D rendering emphasize the presence of epicardial fat (white), with some regions showing visible myocardium on the epicardium (grey voxels). However, the amount of epicardial fat is larger in the ARVC heart than in the healthy heart. Figure 1c displays a cropped volume in the long axis view of both ventricles of the ARVC heart extracted from the red dashed box in Fig. 1b (left image). The three virtual 3D sub-samples corresponding to the ARVC triangle were selected: in the RV free wall (top left), in the apex septum (bottom left), and in the RVOT (right).

**Figure 2:**
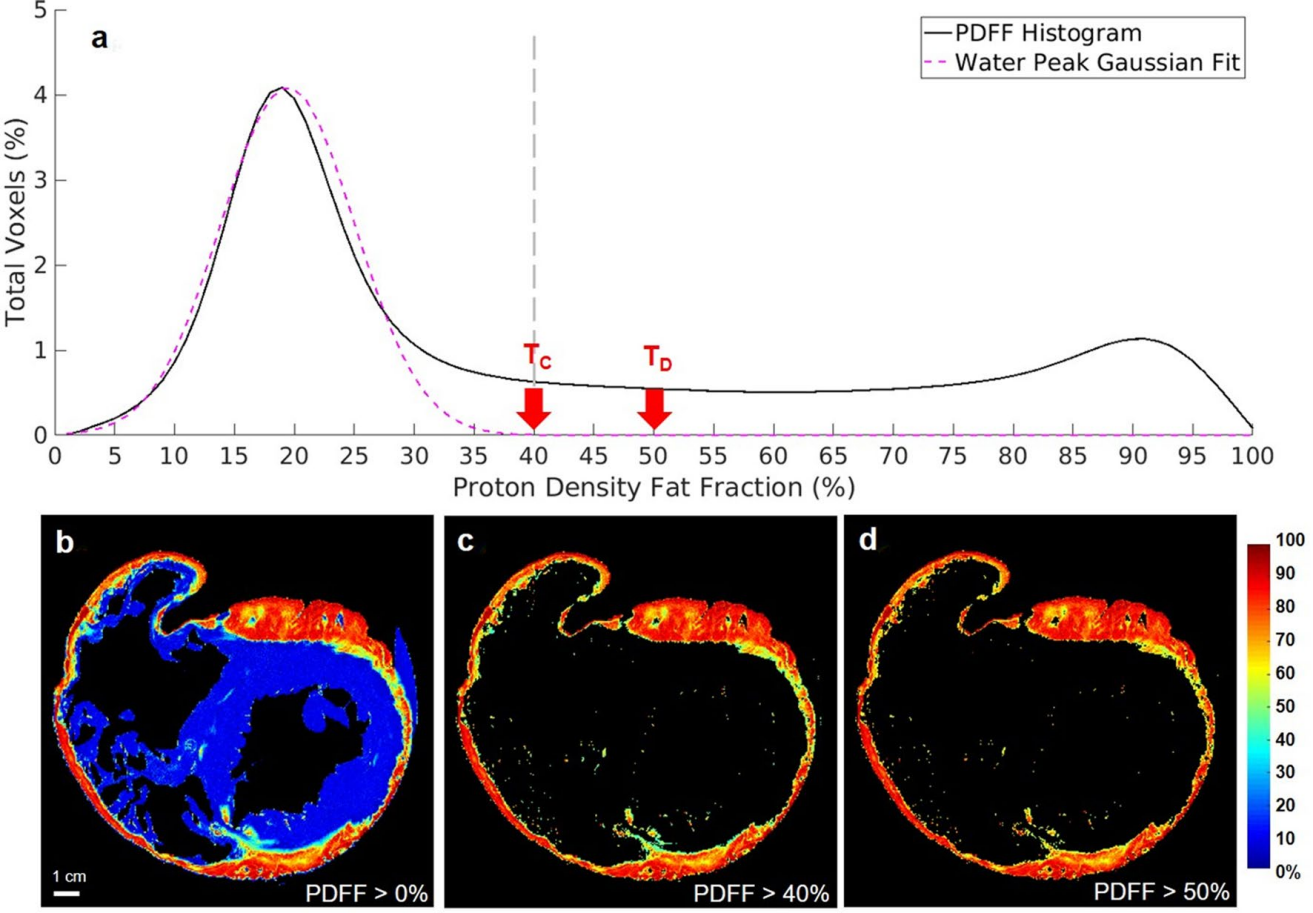
Histogram of the Proton Density Fat Fraction. Histogram of the Proton Density Fat Fraction (PDFF) map (**a**) is extracted from whole human heart. Different thresholds (red arrows) are applied: PDFF > 0% (**b**), PDFF > 40% (T_C_ and **c**) and PDFF > 50% (T_D_ and **d**). The gray dashed line (**a**) corresponds to the delineation between the water peak and the fat.

Figure 2a is a plot of the cumulative histogram from the 3D PDFF reconstructed volume on the ARVC heart (Fig. 1b). Two main peaks are observed, which correspond to water (PDFF at 21%) and fat (PDFF at 92%). The pink dashed line is the result of Gaussian fitting of the water peak. Red arrows indicate thresholds that result from the Gaussian fitting (red arrow T_C_) and Dixon technique (red arrow T_D_). The number of voxels included between both thresholds represents 6.42% of the total number of voxels over the entire volume. Images displayed in Fig. 2 show the same short axis view located in the mid ventricular region of the PDFF without any threshold (Fig. 2b), as well as with the T_C_ (40%) and T_D_ (50%) thresholds defined in Fig. 2b, respectively. Most fat is located in the outer layers of the ventricular wall and images obtained with both thresholds (Figs. 2c and 2d) appear similar. The same cumulative histogram is plotted (See Supplementary Figure S1) for the healthy heart, where the peak corresponding to water is at 17% and the threshold T_C_ determined from the Gaussian function is 41%.

**Figure 3:**
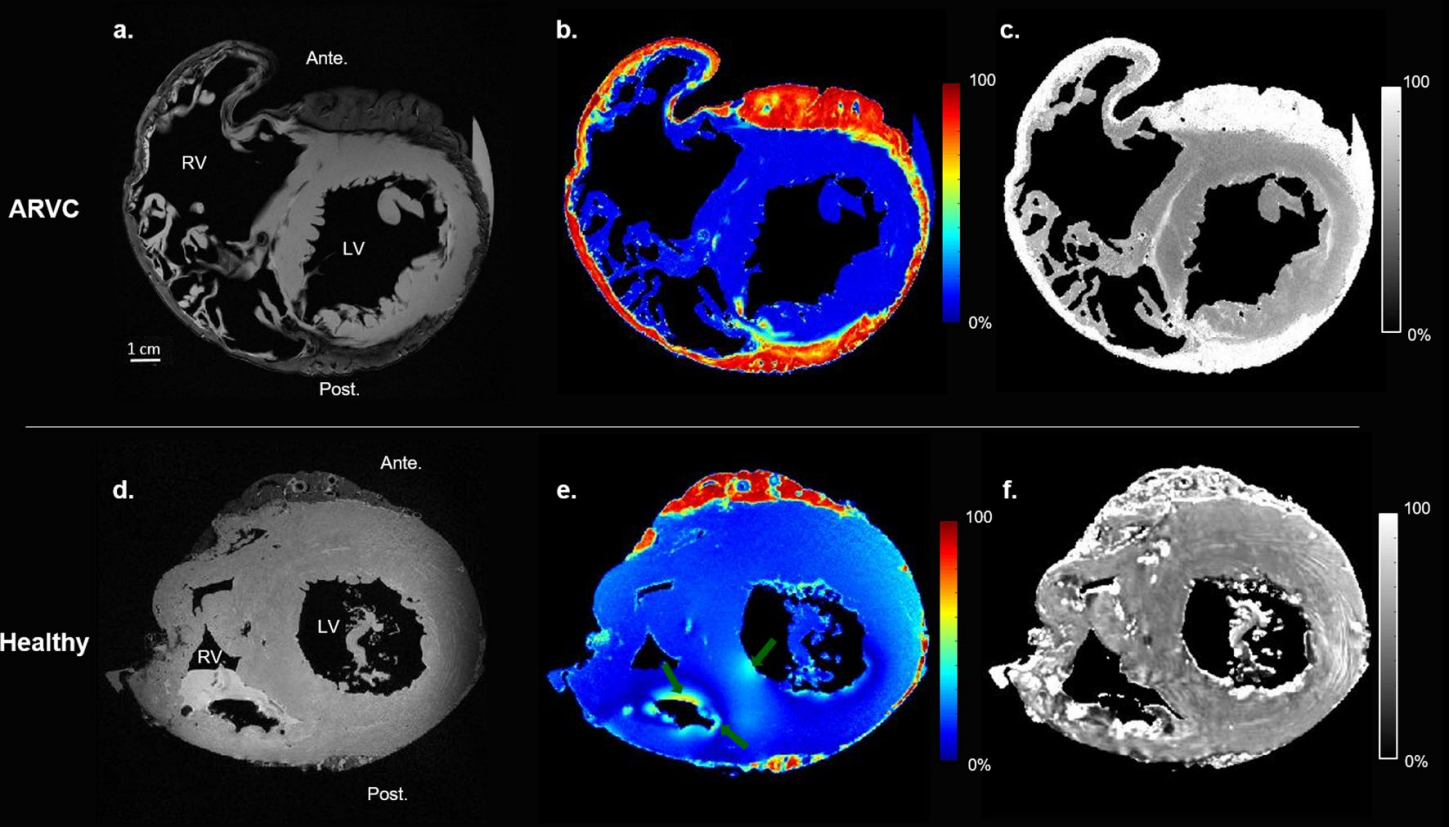
Short axis view of the ARVC and healthy hearts. Short axis view of the ARVC heart (top) and the healthy heart (bottom). An IDEAL first echo (**a**,**d**), proton density fat fraction map (PDFF) (**b**,**e**) and MTR contrast (**c**,**f**) of both the hearts respectively are shown. The healthy heart presents some residual formalin in the RV, leading to some susceptibility artefacts on the PDFF (dark green arrow).

Figure 3 shows a mid-ventricular short axis views of both hearts. The RV of the healthy heart is thicker than the RV of the ARVC heart (Fig. 3a,d) with a low amount of epicardial fat surrounding the RV, and without fat infiltration in the septum (Fig. 3b,e). Moreover, MTR of the healthy heart (Fig. 3f) denotes an absence of bright signal in the myocardium, in contrast to ARVC heart (Fig. 3c).

**Figure 4:**
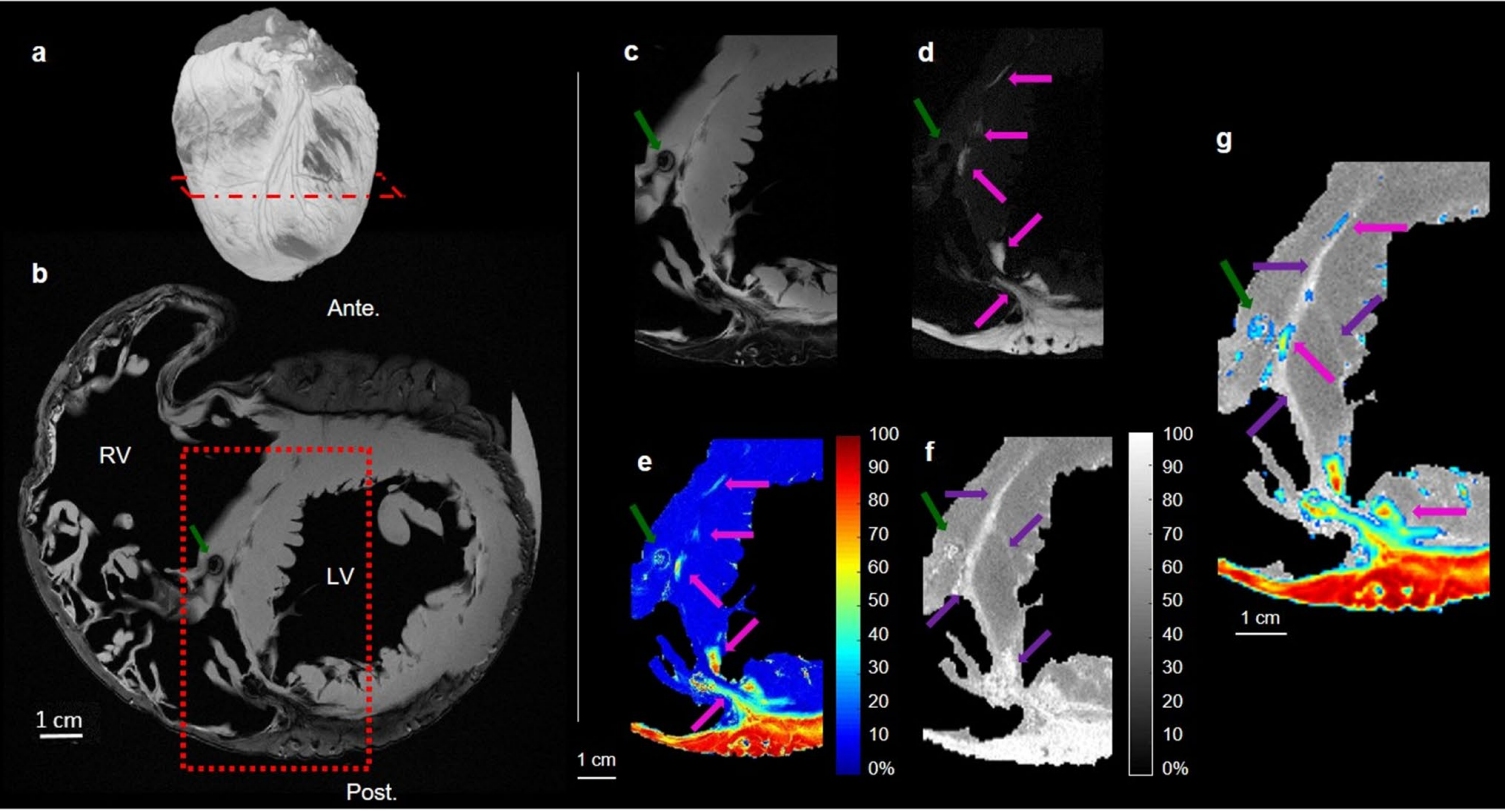
IDEAL outputs and MTR of the ARVC heart. Short axis view of the ARVC heart. A 3D rendering (**a**) showing water in grey, fat in white and the position of the slice in red, IDEAL first echo (**b**), proton density fat fraction map (PDFF) (**e**) calculated from water (**c**) and fat (**d**) images and MTR contrast (**f**) are shown. An overlay of the proton density fat fraction map on the MTR is displayed (**g**). All images show an artefact due to an air bubble trapped during the sample fixation (dark green arrow). (**d**) and (**e**) display fat infiltrations (dark green arrows) and (f) shows the presence of fibrosis (purple arrows).

Figure 4 presents a detailed analysis of the water-fat and MT imaging in the slice presented in Fig. 2. Figure 4a illustrates the slice location on the 3D rendering of the PDFF. Figure 4b depicts the image with the shortest TE in the IDEAL sequence. Fat appears as a hypointense signal surrounding the ventricles, with clear delineation from the myocardium in the left ventricle. The RV free wall is composed of only a thin layer (3–10 mm) of fatty tissue with little or no apparent myocardium. The red dashed rectangle is focused on the interventricular septum. Within this rectangle, there is a susceptibility artefact (dark green arrow) due to a remaining air bubble trapped in the right side of the septum during sample fixation. Figures 4c–g show cropped views of the red dashed rectangle displayed in Fig. 4b. Figures 4c and 3d show water and fat images resulting from hierarchical IDEAL post-processing. Pink arrows in Fig. 4d show fat infiltrations into the septum, which are also observed on the PDFF map (Fig. 4e, displayed without threshold). These adipose tissues are present in smaller quantities (40–70% of fat) than the fat surrounding the ventricle (> 70%). Figure 4f shows the MT ratio map of the same region. A high intensity signal (MTR > 75%) is observed in the septum (purple arrows). Figure 4g shows superimposition of the PDFF (with a 40% threshold) over the MTR image. Fatty infiltrations are observed in the vicinity of the region with a high MTR. This overlaid image indicates that the hyperintense signal in the MTR (purple arrows) does not fully correspond to the presence of fat in some small areas (pink arrows).

### Histological validation

**Figure 5:**
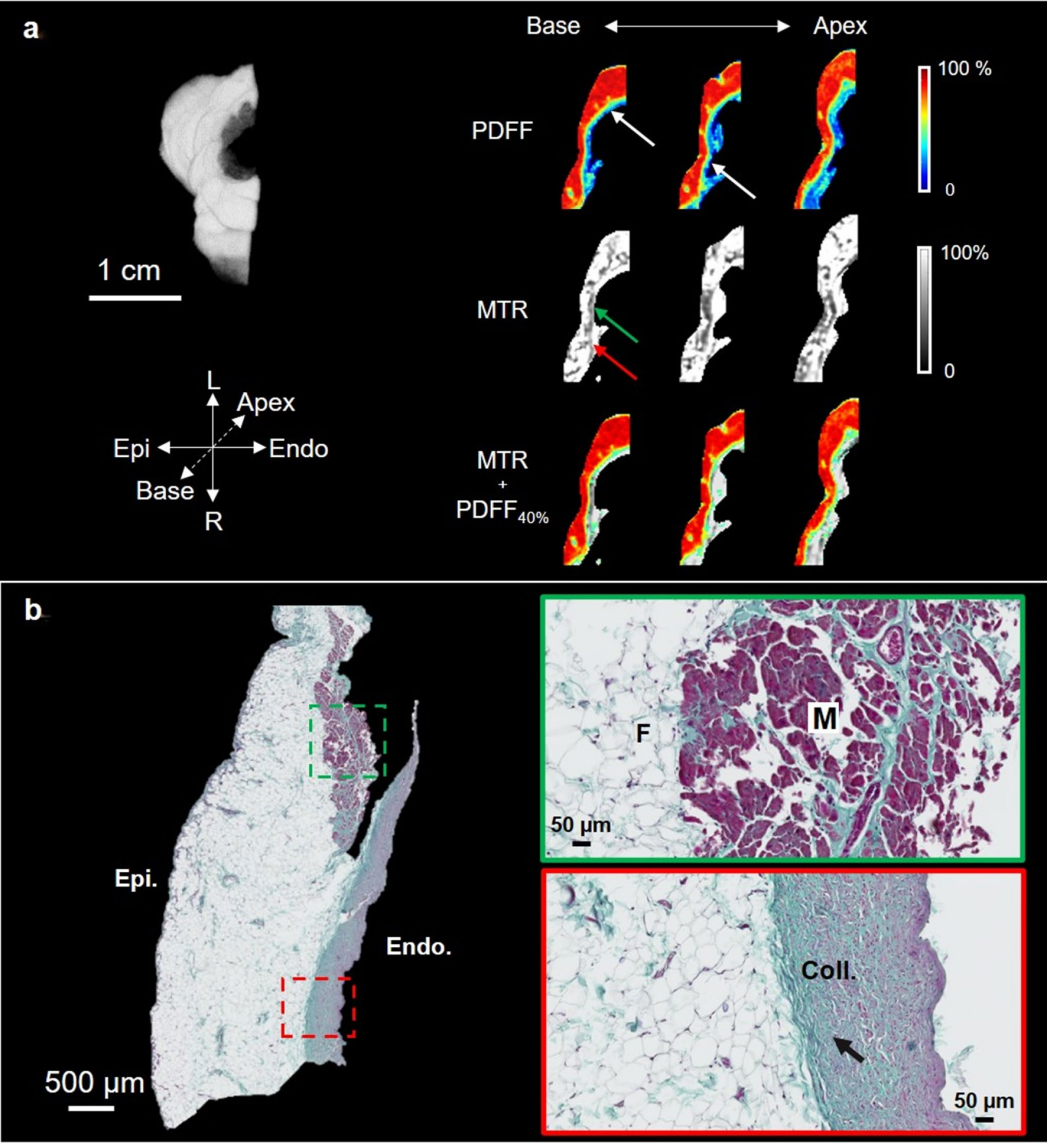
RVOT subsample of the ARVC heart. Axial view (**a**) of the RVOT cut sample (1.4 × 1  ×  0.6 cm^3^). PDFF with epicardial fat contact to the myocardium (white arrows), MT ratio (MTR) and MTR with fat > 40% (MTR + PDFF_40%_) overlaid are presented. All the MRI slices are 200 µm thick and separated by 1 mm. Corresponding histological slices (**b**) stained with Masson’s trichrome are also shown and zoomed regions (green and red dashed rectangles) are displayed. Muscle (M), fat (F) and collagen (Coll.) are visible. The myocardium is filled with collagen (black arrow).

Image processing shown in Fig. 4 was applied in three sub-volumes of the ARVC heart, corresponding to the ARVC triangle; these are presented in Figs. 5 (RVOT), 6 (RV free wall), and 7 (apical RV septum). Figure 5a (left) shows a 3D rendering of the PDFF of the selected subsample. Figure 5a (right) shows 3 slices of 200 µm thickness, separated by 1 mm each; these enable analysis of the transmural profile of an axial view of the PDFF map (top row), the MTR (middle row), and the PDFF with a 40% threshold superimposed MTR (bottom row). The PDFF shows a strong presence of pericardial fat in red (> 80%). The PDFF progressively decreases to 35% when approaching the cardiac muscle; some areas contain mostly fat (white arrows). MTR images of corresponding slices show a homogeneous signal between 80 and 90%, except in some areas in the myocardium where the MTR decreases to 70% (red arrow). Overlay of the two modalities (MTR + PDFF_40%_) shows that this hyperintense signal in the MTR does not correspond to the presence of fat. In the histology image (Fig. 5b left), the ventricular wall is mainly composed of fat, with a thin layer of residual myocytes on the endocardial side. Two areas are magnified (green and red dashed rectangles) and displayed in Fig. 5b (right). In these images, myocytes (M, pink color), fat (F, white color), and collagen (Coll., blue color) are observed in the myocardium in both regions. However, the upper magnified image shows less collagen, compared to the lower magnified image. In the lower magnified image, there is a higher density of collagen (black arrow) in the region near the fat, compared to the region near the endocardium.

**Figure 6:**
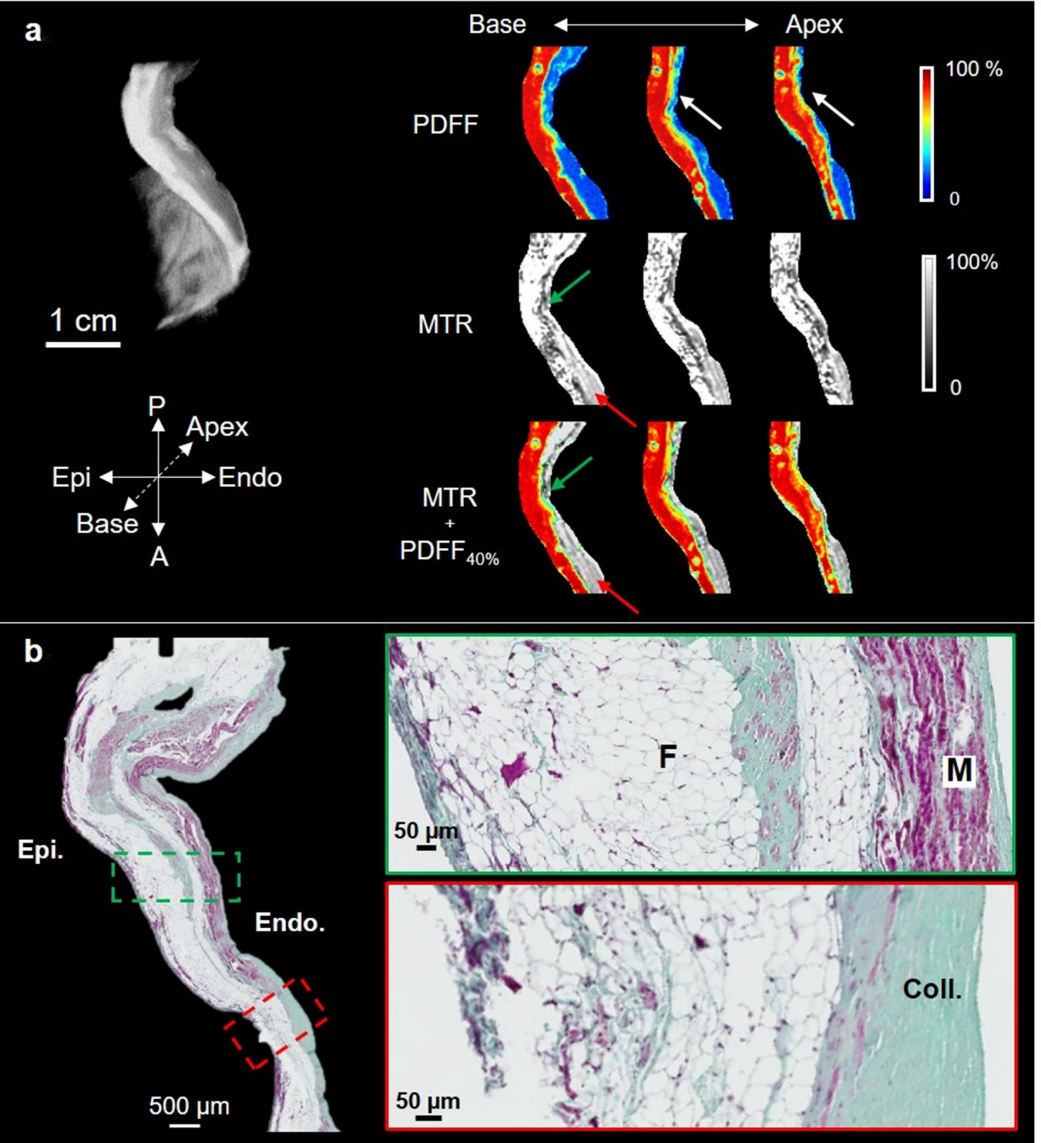
RV free wall subsample of the ARVC heart. Axial (**a**) view of the RV free wall cut sample (2 × 1.5 × 1 cm^3^). PDFF with epicardial fat contact to the myocardium (white arrows), MT ratio (MTR) and MTR with fat > 40% overlaid (MTR + PDFF_40%_) are presented with area of concentrated (red arrows) and spread (green arrows) fibrosis. All the MRI slices are 200 µm thick and separated by 1 mm. Corresponding histological slices (**b**) stained with Masson’s trichrome are also shown and zoomed regions (green and red dashed rectangles) are displayed. Muscle (M), fat (F) and collagen (Coll.) are visible. The myocardium is filled with collagen.

Figure 6a shows a similar analysis in the RV free wall subsample. In the first PDFF image, there is a clear delineation between the epicardial layers, mainly composed of fat (red color), and the endocardium (in blue); some pixels show a progressive transition (pixels in yellow). In the two subsequent images, delineation is more difficult, because most pixels contain fat (white arrows). MTR images (second row) show no MT (ratio close to 100%) in the epicardial fat and a lower ratio in the endocardium, ranging from 85% (red arrow) to 55% (green arrow). Composite images (MTR + PDFF_40%_) confirm that regions with PDFF < 40% (no fat) correspond to areas where MT is present.

In the histological results (Fig. 6b), the left image shows a slice with a heterogeneous composition including different layers of fat, myocytes, and collagen. In the green box, a collagen layer surrounding remaining myocytes is present inside the epicardial fatty infiltration; myocytes are present on the endocardium, surrounded by a thin collagen layer. In the red box, tissue in the endocardium is mainly composed of collagen.

**Figure 7:**
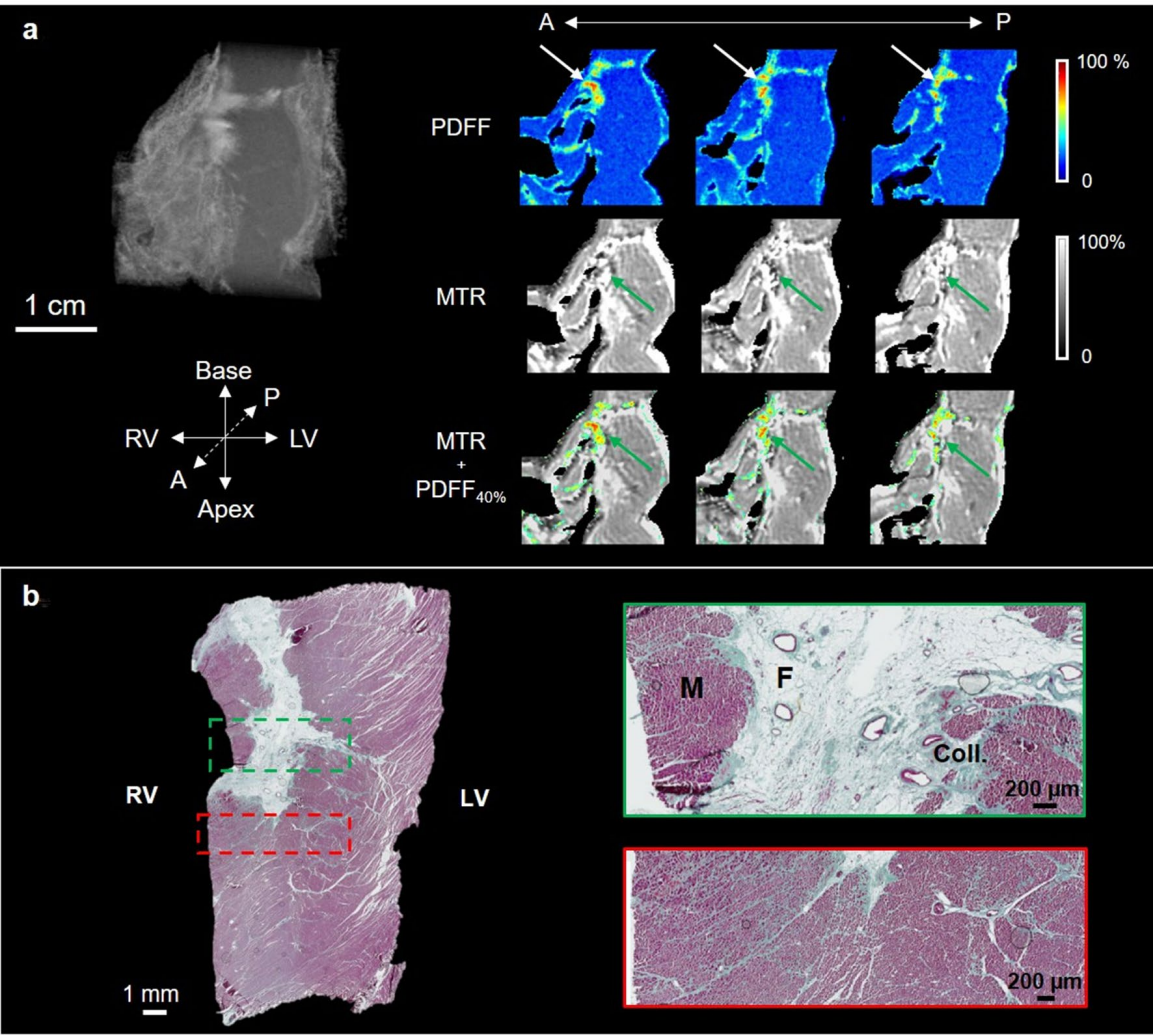
RV apex septum subsample of the ARVC heart. Coronal view of the septum sample (1.9 × 1.8 × 1.5 cm^3^) in the apex region (**a**). PDFF with fat infiltration (white arrows), MT ratio (MTR) with fibrosis areas (green arrows) and MTR with fat > 40% (MTR + PDFF_40%_) overlaid are presented. All the MRI slices are 200 µm thick and separated by 1 mm. Corresponding histological slices (**b**) stained with Masson’s trichrome are also shown and zoomed regions (green and red dashed rectangles) are displayed. Muscle (M), fat (F) and collagen (Coll.) are visible.

A coronal view of the RV septum sample is shown in Fig. 7a, which allows analysis of the transmural profile. The PDFF map (Fig. 7a) shows the presence of fat in the septum (white arrows on first line) with an intense signal in three slices of 200 µm thickness, separated by 1 mm. The center of the fatty infiltration is composed of approximately 80% fat; however, with increasing distance from the center, the fat concentration decreases to 35%. MTR images show a high intensity signal > 90% (green arrows), which decreases to 65% in the muscle. Composite images (MTR + PDFF_40%_) emphasize that regions where PDFF is < 40% (no fat) show a MT ratio > 90% (green arrows); they also show regions with fat. Corresponding histological results are shown in Fig. 7b; the green box highlights a region with high content in adipose tissue, together with the presence of collagen. In the red box, the cardiac muscle is indicated in pink; a large amount of collagen is located close to fatty tissue and layers of collagen are present in cardiac muscle.

Table 1 provides quantitative estimates of fat and tissue contents in ARVC and healthy hearts for the whole hearts and for the three regions of interest of the “ARVC triangle” (Figs. 5–7) through MRI and histological data analysis.

**Table 1:** MRI estimation of total volume of the myocardium, fat and tissue of the ARVC and the healthy hearts in the whole heart and for each region of interest of the “ARVC triangle”. Volumes are calculated from the Proton Density Fat Fraction (PDFF) map with a threshold of 40% for the ARVC and 41% for the healthy hearts. Fat ratios of each regions of interest are calculated based on MRI and histological data.

**ARVC**						
	**MRI**					
	**Total (cm**^**3**^**)**	**Fat (cm**^**3**^**)**	**Tissue (cm**^**3**^**)**	**Fat voxel (#)**	Fat ratio (%)	
**Whole Heart**	339	122.20	216.80	15,277,337	**MRI**	**Histology**
**RVOT**	0.71	0.54	0.17	68,032	76.0	79.60 ± 9.60
**RV Free Wall**	2.85	1.90	0.95	236,912	66.6	61.0 ± 5.60
**RV Apex Septum**	5.15	0.23	4.92	29,318	4.50	7.20 ± 3.80

**Healthy**						
	**MRI**					
	**Total (cm**^**3**^**)**	**Fat (cm**^**3**^**)**	**Tissue (cm**^**3**^**)**	**Fat voxel (#)**	Fat ratio (%)	
**Whole Heart**	306.60	52.70	253.90	6,591,334	**MRI**	**Histology**
**RVOT**	1.85	0.59	1.26	73,735	31.90	39.20 ± 6.30
**RV Free Wall**	3.77	1.08	2.69	135,010	66.6	28.80 ± 7.20
**RV Apex Septum**	4.48	0.06	4.42	7,039	1.30	5.70 ± 2.50

Both hearts had similar volumes (339 cm^3^ vs. 306.6 cm^3^), but with different fat ratios (36.0% vs. 17.2%). Fat ratios according to the MRI data in the RVOT, RV free wall and RV apex septum for the ARVC and healthy hearts were 76.0% vs. 31.9%, 66.5% vs. 28.7% and 4.5% vs. 1.3%, respectively. The histological analysis of the fat ratios in the three regions led to 79.6 ± 9.6% vs. 39.2 ± 6.3%, 61 ± 5.6% vs. 28.8 ± 7.2% and 7.2 ± 3.8 vs. 5.7 ± 2.5% for the ARVC and healthy hearts, respectively.

## Discussion

To the best of our knowledge, this is the first MR study to present high-resolution 3D images of fat and fibrosis obtained by analysis of intact ex vivo human hearts, at an isotropic resolution of 200 µm. Higher spatial resolutions (50 µm isotropic) were reported on hearts from small animals^28,34^. Acquiring 3D images on intact human heart at such a resolution remains challenging in terms of acquisition time, signal to noise ratio and data processing. Moreover, a spatial resolution of 200 µm was considered sufficient to visualize fibro-fatty infiltrations in the myocardium of our specimens. Although performed on ex vivo specimens, the use of dedicated preparation (formalin fixation with gadolinium) and sample imaging conditions (use of fomblin for MR-imaging at high field) is unlikely expected to modify the fat content or fibrosis.

3D fat fraction maps were computed from water and fat images obtained with the IDEAL technique^21,22^ and associated hierarchical IDEAL^27^. This method is preferable to the conventional Dixon^35^ approach, because it is more resilient to the B_0_ inhomogeneity that is inherent to large sample imaging at high-field intensity. Our results showed good agreement between MRI results and histology data in each of the three ARVC triangle regions. As expected, large amounts of fat were observed in the RV free wall and RVOT. Despite the fat content appearing to be lower in the RV apex septum, this particular sample also contained a large part of the LV septum. In the pipeline of image processing, we chose a threshold (T_C_) to discriminate voxels with high fat content based on the PDFF histogram (Fig. 2). In our processing, M_0_ maps of water and fat were obtained after correction of T_1_ and T_2_^*^ values (Eq. 3), derived from a single slice acquisition. This compromise was made to avoid full 3D acquisition of T_1_ and T_2_^*^ maps, which would have required additional processing time. 3D T_2_^*^ maps could have been acquired by using MGE sequences. 3D T_1_ maps were presumed to be more difficult to obtain, due to the inherent difficulty of generating homogeneous flip angles^36^ on a large sample. Thus, a simpler approach was preferred. Correction of the fat fraction map based on averaged T_1_ and T_2_^*^ to achieve the resulting PDFF was not expected to influence the identification of high fat content voxels, as the threshold is calculated from the histogram. The resulting threshold differed from the conventional 50% of the Dixon technique but resulted in a very similar number of voxels attributed to fat (29.8% vs 36.0% for the whole heart, 70.2% vs 76.0% for the RVOT, 57.4% vs 66.6% for the RV free wall and 1.6% vs 4.5% for the RV apex septum, respectively). We report a good correspondence between the fat ratios retrieved from the PDFF and the histological analysis in the three observed regions (Table 1). However, the RV apex septum ratios slightly differs and displays a low fat content. Nonetheless, the differences remained in the range of uncertainty reported on histological analysis.

We also developed an MT acquisition sequence to identify fibrotic areas based on a preparation module with off-resonance pulses, which could saturate a specific region of the proton spectrum containing macromolecules^37^. This approach to detect macromolecules (e.g. collagen) has been used to observe fibrosis in liver^23^, kidney^24,38^, and in intestinal tissues^26^. When combined with the PDFF, this MT acquisition technique allows the identification of areas where the MTR denotes the presence of macromolecules and the PDFF excludes the presence of any fat content. Indeed, comparison with histology findings confirmed the presence of collagen in areas where the MTR was > 70% and PDFF was < 40%. Both of these techniques, when used in combination, help to discriminate areas of fat from areas of fibrosis; they also help to identify areas where fat is already present, and fibrosis is emerging.

Bouazizi et al*.*^39^ presented analyses of left human atria; they compared T_1_ maps with 3-point Dixon-based fat fraction maps to differentiate fibrosis from adipose tissue. This method was proposed to discriminate fibrosis, fat, and fibro-fatty infiltrations in human left atria wall at the tissue level, based on the Dixon method (fat) and analysis of the T_1_ distribution (fibrosis). However, these analyses were not performed in 3D and included 5 slices of 1 mm thickness at 200 × 200 µm^2^ in-plane resolution. In the present study, we proposed 3D isotropic imaging of whole heart at 200 µm. We proposed a different approach for identification of fibrotic tissues using MT; acquisition of 3D T_1_ maps was reportedly challenging when using large samples, due to different sources of error^40–42^. Eliav et al*.*^43^ and Kim et al*.*^44^ demonstrated that the collagen matrix greatly contributes to the MT; moreover, there was a linear correlation between the MTR ratio and percentage of collagen in vitro. It was recently shown that MTR varies in the heart as a function of the collagen type (I vs. III)^45^. Indeed, the relative proportion of type I and type III collagen is expected to evolve with age and pathologies in the cardiac tissue. These factors contribute significantly to the observed cardiac fibrosis^46^. A more thorough analysis of the MTR contrast mechanism for quantifying the fibrosis using this technique is thus required and was considered out of the scope of the present work.

In our study, pathological regions of interest were extracted from whole heart 3D MR acquisition. Given the MRI FoV and resolution, we reported the distances and positions on the ex vivo sample to guide dissection for histology. Our results showed that MRI and histology were in good agreement in terms of both fat quantification and fibrosis localization for each sample within the “ARVC triangle”.

In this pathological heart with ARVC, we found abnormalities in the short axis view, such as RV wall thinning characterized by a loss of cardiac muscle in the whole RV free wall. These abnormalities are consistent with the Task Force Criteria for ARVC^8,47^. Indeed, a loss of > 40% of myocytes with fibrous replacement of the RV in the free wall is a major criterion for tissue characterization, consistent with the proportion of 66.6% fat (Table 1) observed in the RV free wall. In some regions of the free wall, fat entirely replaced the myocardium; in other regions, fibrosis filled and replaced the myocardium in the vicinity of fatty infiltrations identified in both MRI and histology. In the RV apex septum, fatty infiltration and fibrotic tissue were observed (Fig. 7). In the RV septum, the percentage of fat was much lower than in the other regions (Table 1), consistent with the findings of previous studies, in which the RV septum was less involved in patients with ARVC^9,10^. The RVOT exhibited the highest fat content (Table 1), together with a high fibrosis content, as determined by MT (Fig. 5). These results were consistent with the ARVC pathology. For comparison, the fat content of the healthy heart was 17.2%, roughly half of the value observed in the ARVC heart (36.0%).

An additional animated GIF file presents the PDFF and MT ratio volumes covering the whole ARVC heart (See Supplementary Fig. S2). Some regions in the LV exhibited fat and fibrosis in the posterolateral wall of the sub-epicardial regions, consistent with in vivo imaging data reported in prior studies^48–51^.

This study had several limitations. First, only two hearts were included, inherent to the difficulty to access such unique intact whole hearts. Although MT and fat fraction imaging are technically feasible in vivo, achievement of the necessary spatial resolution in vivo represents a significant technical challenge. The strong efforts of the MRI community toward 3D in vivo imaging of the heart is expected to result in more spatially resolved images. Some studies have reported the development of intra-cardiac MRI with catheter micro-coils to increase the image spatial resolution in restricted field of view and the cardiac gating to remove movement artefact^52,53^. This can open the possibilities to apply IDEAL and MT to such a resolution that fibro-fatty infiltrations could be observed in clinical situations.

## Conclusions

In this study, the fat fraction map combined with MT imaging provided 3D volumes at an isotropic resolution of 200 µm, allowing to differentiate healthy myocardium, fat, and fibrosis. Data from MRI showed good agreement with those from histological analysis. This non-destructive approach allows the investigation of potential alterations of the whole human heart in pathologies where fibrosis and/or fat infiltrations are expected to occur.

## Supplementary Information

Supplementary Figures.

## Data Availability

The datasets generated during and/or analysed during the current study are not publicly available due to restrictions on human donor data.

## Abbreviations

ARVC: Arrhythmogenic right ventricular cardiomyopathy
FoV: Field of view
GRAPPA: Generalized autocalibrating partial parallel acquisition
IDEAL: Iterative decomposition of water and fat with echo asymmetry and least-squares estimation
LV: Left ventricle
MGE: Multiple gradient echo
MT: Magnetization transfer
MTR: Magnetization transfer ratio
NA: Number of averages
PDFF: Proton density fat fraction
RARE: Rapid acquisition with relaxation enhancement
ROI: Region of interest
RV: Right ventricle
RVOT: Right ventricle outflow tract
TE: Echo time
TR: Repetition time
