# Supplementary material for: A 3D high resolution MRI method for the visualization of cardiac fibro-fatty infiltrations
Source: Sci Rep. 2021 Apr 29;11:9266. doi: 10.1038/s41598-021-85774-6 (PMC8084928; doi:10.1038/s41598-021-85774-6)
Supplement: Supplementary file 1 — Supplementary Figures. [file 41598_2021_85774_MOESM1_ESM.docx]

**Supplementary Materials**

**A 3D high resolution MRI method for the visualization of cardiac fibro-fatty infiltrations.**

K. Haliot^1,2,3^, V. Dubes^1,2,3^, M. Constantin^1,2,3^, M. Pernot^4^, L. Labrousse^1,4^, O. Busuttil^4^, R. D. Walton^1,2,3^, O. Bernus^1,2,3^, J. Rogier^4^, K. Nubret^4^, P. Dos Santos^1,2,3,4^, D. Benoist^1,2,3^, M. Haïssaguerre^1,2,3,4^, J. Magat^1,2,3^, B. Quesson^1,2,3^

^1^ IHU Liryc, Electrophysiology and Heart Modeling Institute, Fondation Bordeaux Université, F-33600 Pessac-Bordeaux, France

^2^ Univ. Bordeaux, Centre de recherche Cardio-Thoracique de Bordeaux, U1045, F-33000, Bordeaux, France

^3^ INSERM, Centre de recherche Cardio-Thoracique de Bordeaux, U1045, F-33000 Bordeaux, France

^4^ Bordeaux University Hospital (CHU), F-33600 Pessac, France

**
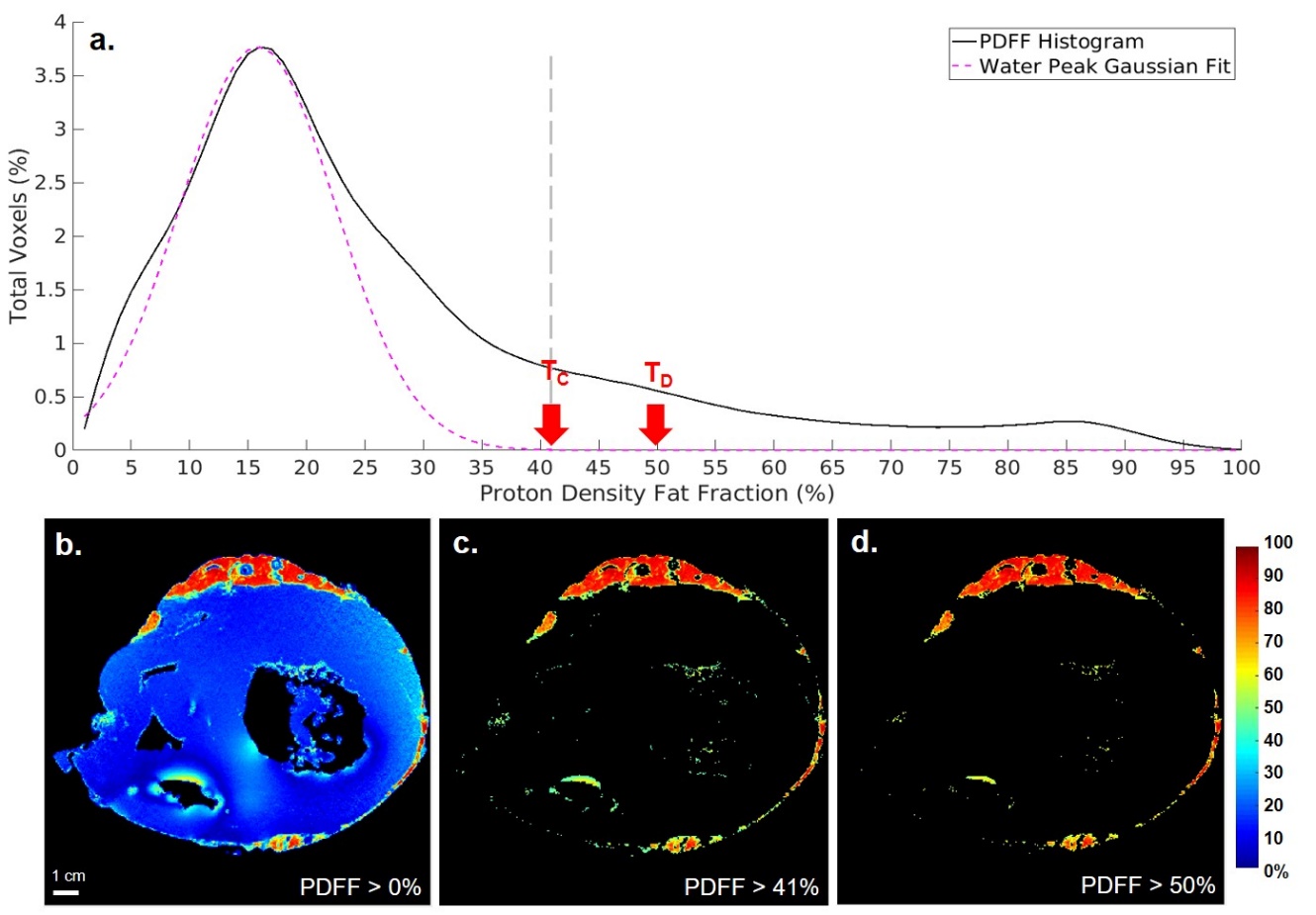
**

**Supplementary Figure S1 – Histogram of the Proton Density Fat Fraction of the healthy heart.** Histogram of the Proton Density Fat Fraction (PDFF) map of the healthy heart (a) is extracted from whole human heart. Different thresholds (red arrows) are applied: PDFF > 0% (b), PDFF > 41% (TC and c) and PDFF > 50% (TD and d). The gray dashed line (a) corresponds to the delineation between the water peak and the fat.


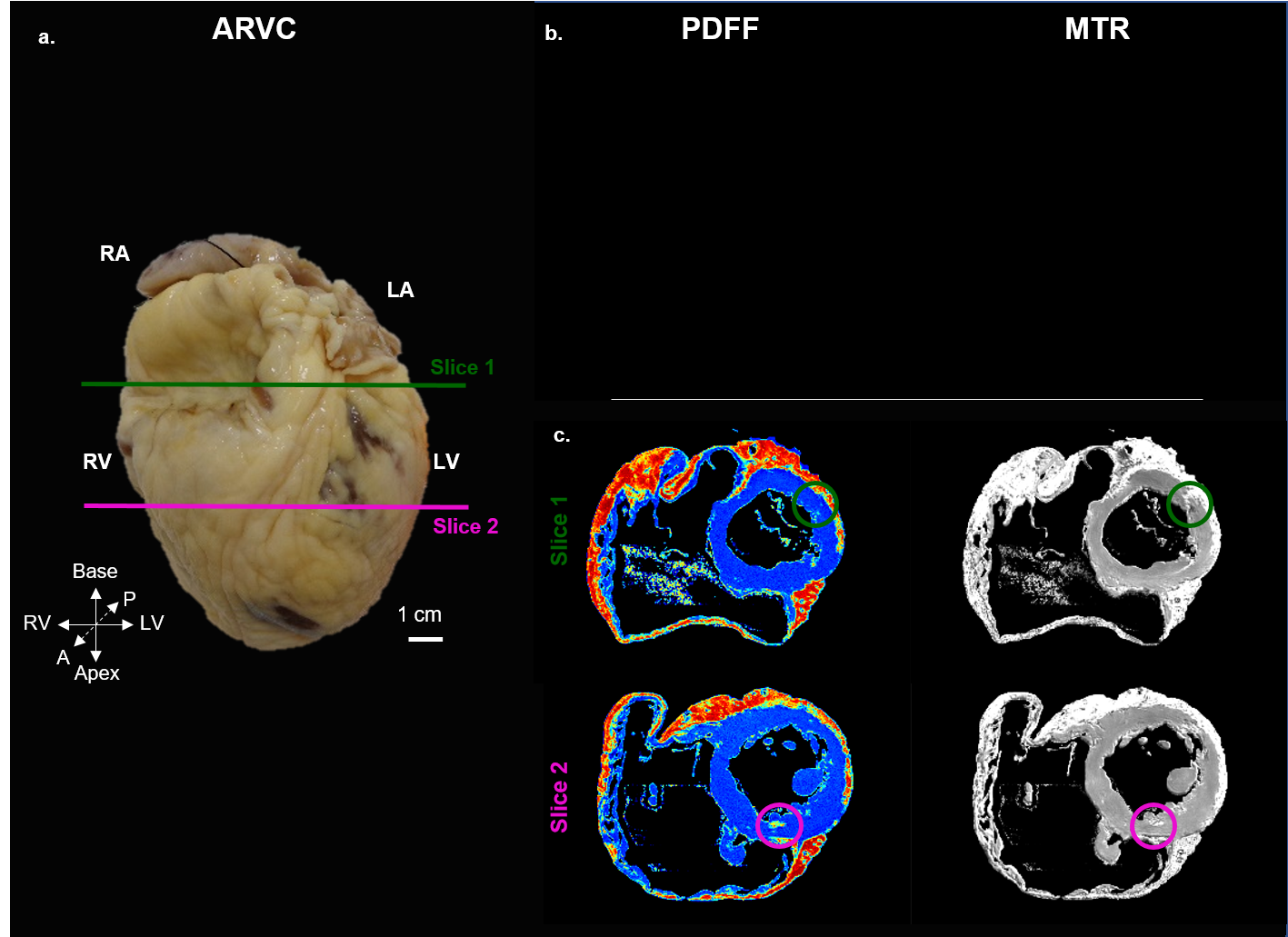


**Supplementary Figure S2 – Animated GIF of the ARVC heart PDFF and MTR volumes.** A photograph of the ARVC heart is displayed (a). PDFF (b left) and MTR (a right) volumes covering the whole ARVC heart are presented as an animated GIF (GIF is available for downloading separately). Some regions in the LV exhibit fat and fibrosis in the posterolateral wall of the sub-epicardium and 2 slices are represented (c). Slice 1 shows the presence of fibrosis on MTR in the LV free wall (green circle) which is not associated with fat as shown on the PDFF (green circle). The PDFF on slice 2 shows a fat infiltration (pink circle) and the MTR shows a hypersignal (pink circle).
